# The mediating role of sleep quality in the relationship between orthorexia nervosa and fear of COVID-19

**DOI:** 10.1038/s41598-023-42825-4

**Published:** 2023-09-19

**Authors:** Satı Dil, Tuğba Yıldırım, Pınar Çiçekoğlu Öztürk

**Affiliations:** 1 Faculty of Health Sciences, Universtiy of Çankırı Karatekin, Çankırı, Turkey; 2 Fethiye Faculty of Health Sciences, Universtiy of Muğla Sıtkı Koçman, Muğla, Turkey

**Keywords:** Risk factors, Psychology, Human behaviour

## Abstract

The COVID-19 (SARS-CoV-2) pandemic has jeopardized humanity worldwide and has considerably altered the healthy lifestyle behaviors of societies. This study examined the possible mediating role of sleep quality in the relationship between orthorexia nervosa and fear of COVID-19 among Turkish adults. This study used an observational and descriptive design. It was carried out online with 1,130 participants across Turkey between April and August 2021. The data were collected using a questionnaire, the Fear of COVID-19 Scale (FC-19S), the Pittsburgh Sleep Quality Index (PSQI), and Orthorexia Nervosa-R (ON-R). The participants had an ON-R mean score of 3.08 ± 0.90 and a mean PSQI score of 8.03 ± 2.80. Likewise, their mean FC-19S score was 18.24 ± 6.55. There was a significant positive correlation between the FC-19S and the PSQI (r = 0.208; p = 0.000) as well as a significant negative correlation between the ON-R and the PSQI (r = − 0.145; p = 0.000). In addition, the results of the process model analysis supported our hypotheses that the PSQI was a predictor of ON-R and that its direct and indirect effects were moderated by FC19S and the PSQI. A one-unit increase in FC-19S scores causes an average 1% increase on the ON-R scale, while it generates an 8% increase on the PSQI. It was determined that the PSQI total score caused an average of 6% increase in the ON-R scale score. The findings of this study showed that sleep quality has a mediating role in the relationship between orthorexia nervosa and fear of COVID-19 experienced by Turkish adults during the pandemic. For this reason, governments need to take the necessary precautions concerning this subject when creating action plans for possible global crisis situations that may adversely affect public health in the future.

## Introduction

Throughout history, humans have faced various infectious diseases, such as plague, Severe Acute Respiratory Syndrome (SARS), and Ebola. In the last few years, COVID-19, which has become an important public health problem by affecting all areas of life, has taken first place in death, disease, and contagiousness rates^1^. Therefore, numerous countries have imposed several restrictions, have provided isolation and treatment services for infected persons, have followed up with contacts, and have applied various protective and preventive (wearing masks, vaccination, curfew, social distancing, regular hand hygiene, etc.) measures for the sake of public health and safety. Furthermore, the rates of positive cases and deaths announced in the media, speculations about treatment methods and vaccines, the uncertainty brought by lockdowns, and information pollution have all caused emotional exhaustion and intolerance of ambiguity, fear, depression, and anxiety^2,3^. Fear is known in the literature as a basic emotion activated in response to perceived threats and a defense mechanism that increases one’s chances of survival^4^. However, fear can be a source of motivation for positive behavior change, and it can also cause maladaptive behaviors that prevent the person from making logical decisions to protect himself^5,6^. Studies have shown that the fear of COVID-19 causes positive behavioral changes (nutrition and hygiene, mask, social distance protection, etc.) in individuals to protect themselves and their environment^7^. and negative situations such as suicide, sleep disorders, decreased physical activity, negative body image, smartphone and social media addiction and social isolation^8–12^.

Changes in the nutritional patterns of individuals due to the fear of COVID-19 have emerged as behaviors such as directing to hypercaloric diets, spending too much time preparing their food, or regularly turning to take-out services^13,14^. Tragantzopoulou^15^ suggested that people’s fear of COVID-19 puts them at risk of becoming obsessed with healthy eating, especially due to isolation or following restrictive diets that focus on boosting immunity. Kusnierz et al.^16^ discovered that disruptions to people’s daily routines alongside restrictions on outdoor activities can worsen people’s weight and shape concerns, adversely affect eating, exercise, and sleeping patterns, and put them at risk of developing orthorexia and other eating disorders. Orthorexia Nervosa (ON), first defined by Bratman^17^ as an “obsession with healthy eating”, is a pathological obsession whereby one avoids foods they perceive to be “unhealthy” or “dirty.” As a trait, ON is characterized by restrictive and avoidant eating behavior and a tendency toward pathological obsession and preoccupation with healthy, strictly organic, and biologically pure foods^18^. ON focuses on the quality of food consumed rather than its quantity^19^, and what is characteristic of ON is a gradual intensification of imposed dietary restrictions^20^. Meals are prepared with the utmost care and attention, and any deviation from the imposed norms leads to a feeling of fear, guilt, shame, and further dietary restrictions^21–23^. Hence, ON can cause weight loss and nutritional deficiencies. In this context, one might describe it as “an unhealthy addiction to healthy eating”^24^. However, the Diagnostic and Statistical Manual of Mental Disorders (DSM-5)^25^ has not yet officially recognized it.

Changes in sleeping patterns constitute another behavioral change that the fear of COVID-19 has caused^12^. Sleep quality is a key indicator of health, and a fundamental aspect of the sleep–wake cycle is circadian rhythm^26^. Daylight synchronizes the circadian rhythm. Other stimuli that affect the sleep–wake cycle include one’s social life, working hours, meal times, physical activity, temperature, and melatonin levels^27^. Sleeping late due to emotional turmoil (as induced by the lockdown), oversleeping in the morning, and the opportunity to sleep all day long all alter people’s circadian rhythm and cause many to develop insomnia, excessive drowsiness, or both^28^. Numerous studies have reported contracting COVID-19 and anxiety about coming in contact with it and that it is life-threatening and lacks definitive treatment, leading many to develop sleep disorders—particularly insomnia^29–31^. According to the 2023 data from the World Health Organization, the number of COVID-19 cases in Turkey is 17,004,677, and the number of deaths is 101,419^32^. These figures (rates) are worrisome for Turkey, which has a mostly young-middle-aged population. In addition to death and illness rates, COVID-19 has brought many problems, such as anxiety^33,34^, depression^35,36^, and sleep and nutrition disorders^29,37–39^, that significantly affect people’s quality of life^40,41^, and this has led researchers to focus on these issues. Although there have been many studies on fear of COVID-19, eating behaviors, and sleep quality in Turkey, it is still an unanswered question what the mediating roles are in the relations between these variables. It is very important to identify the factors that play a role in this relationship, to increase the awareness of adults in Turkey about the importance of healthy lifestyle behaviors, and to take steps towards solving the problems associated with fear of COVID-19 and orthorexia.

For this purpose, in this study, an answer was sought to the question of whether sleep quality has a possible mediating role in the relationship between fear of COVID-19 and orthorexia nervosa in Turkish adults.

**Figure 1:**
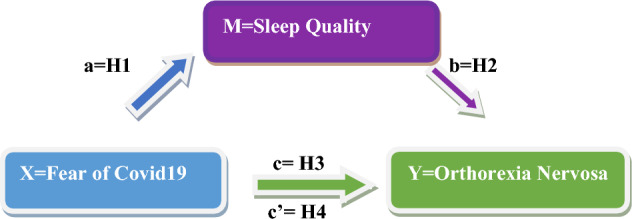
Research model and hypotheses.

Figure 1 shows the theoretical model and hypotheses of the study based on the correlational relationship between the variables.

### Hypothesis

#### H1

Level of fear of COVID-19 is associated with sleep quality. (a way).

#### H2

Level of sleep quality is associated with orthorexia nervosa. (b way).

#### H3

Level of fear of COVID-19 is associated with orthorexia nervosa. (c way).

#### H4

Level of sleep quality is the mediating variable considering the correlation between the level of fear of COVID-19 and orthorexia nervosa. (c’way).

## Methods

### Study design

This study used an observational and descriptive design and examined the mediating role of sleep quality in the relationship between orthorexia nervosa and fear of COVID-19 among Turkish adults.

### Setting

The research was conducted online through Google Forms between April 22 and August 8, 2021. The snowball sampling method is used in the research.

### Participants

For participant sampling, established criteria for inclusion and exclusion were used. The inclusion criteria were (a) volunteering to participate in the research; (b) no problem with verbal communication; (c) living in Turkey; (d) being between the ages of 18–65; and (e) having an Instagram, Facebook, or WhatsApp account. The exclusion criteria were (a) having a history of psychiatric illness; (b) currently receiving psychiatric treatment, and (c) having active COVID-19 during the data collection process.

### Measures

The data were collected using a questionnaire form prepared by the researcher, the Fear of COVID-19 Scale (FC-19S), the Pittsburgh Sleep Quality Index (PSQI), and Orthorexia Nervosa-R (ON-R).

### Questionnaires

A questionnaire was prepared by the researchers based on studies by Alimoradi et al.^30^, Fu et al.^31^, and Targa et al.^42^. It featured 22 questions that asked the participants about sociodemographic characteristics such as age, gender, marital status, presence of children, education level, employment status, weight, height, weight changes during the pandemic, whether they were infected with COVID-19, death of a first-degree relative from COVID-19, history of chronic illness (hypertension, allergic disorders, asthma, diabetes, joint and rheumatism, etc.), history of chronic illness in first-degree relatives, whether they lived with their family during the pandemic and pre- and postpandemic eating habits. The body mass index (BMI) of the participants was measured based on the WHO’s classification system and involved taking their height and weight. People with BMIs of < 16 are deemed anorexic. Those with BMIs between 16.5 and 18.5 are deemed slim, and those between 18.5 and 25 are deemed ideal weight. People with a BMI between 25 and 30 are overweight. If they have a BMI between 30 and 35, they are class I (moderate) obese. Similarly, if they have a BMI between 35 and 40, they are class II (highly) obese. People with a BMI of > 40.0 are morbidly (or massively) obese^43^.

### The Fear of COVID-19

The Fear of COVID-19 Scale (FC-19S) is a seven-item scale designed to measure the extent to which people are afraid of negative outcomes of the global COVID-19 pandemic^44^. It consists of statements concerning different symptoms of fear caused by the COVID-19 virus. Participants assess how strongly they agree with said statements on a scale from 1 (strongly disagree) to 5 (strongly agree), and the final score is calculated by adding up each score. The scale's minimum and maximum scores are 7 and 35, respectively. Higher scores indicate an intensified fear of COVID-19. In the original study, the FC-19S exhibited good internal consistency, with a Cronbach’s α of 0.82. The reliability coefficient of the current sample was even higher, with a Cronbach's alpha of 0.88^45^.

### Orthorexia Nervosa-R

The first scale used for the measurement of orthorexia nervosa (ON) was Orto-15. This scale was developed by Donini et al.^46^. as a valid self-assessment tool for ON, and its cultural adaptation to Turkish was made by Arusoğlu et al.^47^. After that, the Ortho-15 tool was revised, and the Orto-R scale was prepared. Orthorexia was measured with a 6-item Orthorexia Nervosa-R (ON-R) questionnaire, with a response to the Likert scale ranging from Never to Always^48^. The scale's minimum and maximum scores are 1 and 5, respectively, and higher scores highlight more ON tendencies. Cronbach’s α in the previous study was 0.81 for the total ON-R. In the present study, Cronbach’s α = 0.78 for the ON-R score.

### Sleep quality

The Pittsburgh Sleep Quality Index (PSQI) was developed by Buysse et al.^49^, and Ağargün et al.^50^ conducted a Turkish validity and reliability study. Its internal consistency coefficient was 0.80. It is a self-report scale with 24 items and assesses the sleep quality of a given person within the past month. The first 19 items are self-assessment questions. The remaining five questions are to be answered by the subject’s partner or roommate but are not included in the scoring. The first 19 questions are to be answered by the respondent and assess factors associated with sleep quality, including sleep duration, sleep latency, and the frequency and severity of particular sleep-related disorders. Each item is worth between 0 and 3. One can earn a total score of between 0 and 21 points. A PSQI total score of 5 or higher indicates poor sleep quality. Cronbach’s α in the previous study was 0.80 for the total PSQI^50,51^. In the present study, Cronbach’s α = 0.73 for the PSQI score.

### Application

An online questionnaire was used for a self-report survey. The invitation to participate in the study was posted on Instagram, Facebook, or WhatsApp from 22 April to 8 August 2021. Online informed consent was obtained from all participants in the study. In the online consent form, detailed information was given about the aims, methods, confidentiality, and anonymity guarantees, the voluntary basis of participation and withdrawal, and the possible risks and benefits of participating in the study. The participants were informed that if they filled out the data collection form, this meant that they gave consent to participate in the study. The data collection form is arranged in such a way that one cannot proceed to the next question without answering any of the questions in the form, and the questions are answered only once from the same IP address. The participants were allowed to fill out the data collection tools only once.

A total of 1203 participants participated in the study, but 73 participants were excluded from the study due to having a psychiatric disease history (26), still receiving psychiatric treatment (14), and having active COVID-19 during the data collection process (33). Ultimately, the study included 1130 individuals who completed the online questionnaires.

### Statistical analyses

To avoid bias in the research, the analysis of the data was performed by a statistician independent of the research. Statistical analysis of the study data was performed using SPSS 22.0 software. The effect of mediating variables was examined using the “Process Macro 3.5 Model 4” developed by Hayes^52^. Before proceeding to data analysis, pretests were performed to examine the characteristics of the scales used, the assumption of normality, and the correlation estimates between study variables. Normality, kurtosis, and skewness scores and their cut-off values were examined. Parametric tests were used for further statistical analysis since the scales did not show marginal deviation from the normal curve, the sample size was large and the value of ± 2 was acceptable. Explanatory factor analysis (EFA) was used to determine the factor structure underlying behaviors that cannot be measured directly. To measure the reliability of the scales, reliability analysis was carried out taking into account the Cronbach alpha coefficient^45^. Then, Pearson correlation analysis was performed to explore the relationships between the descriptive statistics of the factors and the variables of the study, which emerged as a result of the factor analysis applied to the scales (Table 1). Within the framework of the created model (Fig. 1), analyses were performed to determine the relationships and mediation between the variables. The results obtained from the model were interpreted using standardized path estimate (β) scores and explained variance (R2) values. In the bootstrap method, deviation-corrected confidence intervals at a 95% significance level were established for indirect effects using 5,000 replication samples. For the indirect effect, if the confidence interval is above or below zero, this supports that the mediation effect is significant^52,53^.

**Table 1:** Descriptive statistics, normality tests, and reliability coefficients of the measured variables. (n = 1130).

Scales	Mean	SD	Median	Min	Max	Skewness	Kurtosis	Shap. Wlk	p
FC-19S	18.24	6.55	18.00	7.0	35.0	0.246	− 0.681	0.978	0.00
PSQI	8.03	2.80	7.00	3.0	19.0	0.700	0.093	0.950	0.00
ON- R	3.08	0.90	3.16	1.0	5.0	− 0.264	0.275	0.984	0.00

SD: standard deviation, FC-19S: fear of COVID-19, PSQI: Pittsburgh Sleep Quality Index, ON-R: Ortorexia Nervosa-R.

## Ethics approval

This study was performed in line with the principles of the Declaration of Helsinki. Approval was granted by the University of Çankırı Karatekin Human Research Ethics Committee [Ethics reference date: 12.02.2021 Number: 19] after the Ministry of Health granted permission for Scientific Research.

## Results

### Prevalence and demographic characteristics

The mean age of the participants was 31.43 ± 10.38. A total of 72.3% were female, 27.7% were male, 55.0% were single, and 78.8% had a bachelor’s degree or higher. A total of 56.8% of the participants worked online, face-to-face, or both throughout the pandemic. A total of 59.4% had no children. A total of 51.3% earned an income equivalent to their expenditures. A total of 15.5% suffered from chronic diseases, 20.2% had COVID-19, and 19.1% had at least one first-degree relative who died from COVID-19. A total of 4.6% (52 people) had a BMI of < 18.5, i.e., they were slim. A total of 58.7% (663 people) were of normal weight, 27.4% (310 people) were obese, and 9.3% (105 people) were class I obese.

### Descriptive statistics

In the first step of statistical analyses, descriptive statistics were computed, and distributions of variables were examined. Values of skewness and kurtosis exceeded the absolute value of + 1 for two of the variables but did not exceed ± 2, as shown in Table 1.

### Group comparisons

Table 2 shows distributions according to some sociodemographic characteristics of scores of FC-19S, PSQI, and ON-R of participants. In these comparisons, it was determined that women's COVID-19 fear level and orthorexia tendencies were statistically higher than men's (p < 0.01). The sleep quality of those who were single and had children was worse (p < 0.01), and those who did not work in any job had higher orthorexia tendencies than those who worked. Those who experienced weight change during the pandemic, those who had a chronic disease both in themselves and their first-degree relatives, and those with low income had more fear of COVID-19, orthorexia tendencies, and poor sleep quality than other groups (p < 0.01), and those who lost a first-degree relative due to COVID-19 had poorer sleep quality.

**Table 2:** Differences in FC-19S, PSQI and ON-R between characteristics of participants (n = 1130).

Characteristics of participants		N	Mean ± SD		
			FC-19S	PSQI	ON- R
Gender	Female	817	19.12 ± 6.59	8.12 ± 2.82	3.13 ± 0.87
	Male	313	15.96 ± 5.84	7.79 ± 2.74	2.94 ± 0.96
	t		7.425**	1.786	3.264**
	p		**0.000**	0.074	**0.001**
Marital status	Single	622	17.96 ± 6.37	8.27 ± 2.85	3.09 ± 0.92
	Married	508	18.59 ± 6.74	7.75 ± 2.71	3.07 ± 0.88
	t		− 1.612	3.110**	0.398
	p		0.107	0.002	0.691
Having children	Yes	459	18.51 ± 6.83	7.75 ± 2.77	3.05 ± 0.87
	No	671	18.06 ± 6.34	8.22 ± 2.81	3.10 ± 0.91
	t		1.142	− 2.806**	− 0.907
	p		0.254	0.005	0.365
Working Status	Yes	642	18.21 ± 6.41	7.92 ± 2.77	3.02 ± 0.89
	No	488	18.28 ± 6.72	8.18 ± 2.84	3.15 ± 0.91
	t		− 0.196	− 1.590	− 2.433*
	p		0.845	0.112	**0.015**
Chronic illness	Yes	175	19.49 ± 7.14	8.90 ± 3.18	3.21 ± 0.85
	No	995	18.01 ± 6.41	7.87 ± 2.70	3.06 ± 0.90
	t		2.746**	4.509**	2.068*
	p		**0.006**	**0.000**	**0.039**
Chronic illness in first-degree relatives	Yes	454	18.84 ± 6.70	8.40 ± 3.02	3.16 ± 0.92
	No	633	17.78 ± 6.47	7.74 ± 2.59	3.03 ± 0.89
	t		2.621**	3.854**	2.384*
	p		**0.009**	**0.000**	**0.017**
Weight change in the pandemic	Yes	679	18.64 ± 6.71	8.54 ± 2.94	3.19 ± 0.88
	No	451	17.64 ± 6.24	7.27 ± 2.40	2.91 ± 0.90
	t		2.524*	7.591**	5.205**
	p		**0.012**	**0.000**	**0.000**
Loss of a first-degree relative due to COVID-19	Yes	216	18.56 ± 7.42	8.42 ± 2.97	3.10 ± 0.91
	No	914	18.16 ± 6.32	7.94 ± 2.76	3.08 ± 0.90
	t		0.797	2.245*	0.325
	p		0.425	0.025	0.745
Income	low income	300	18.63 ± 7.21	8.77 ± 2.97	3.14 ± 0.94
	income equal to expense	579	18.51 ± 6.30	7.79 ± 2.69	3.04 ± 0.88
	high income	251	17.17 ± 6.17	7.71 ± 2.70	3.10 ± 0.87
	F		4.395*	14.485**	1.234
	p		**0.013**	**0.000**	0.292

*FC-19S* fear of COVID-19, *PSQI* Pittsburgh Sleep Quality Index, *ON-R* Ortorexia Nervosa-R.

*p < 0.05, **p < 0.01.

Significant values are in bold.

### Factor and reliability analysis

Before examining the mediation effect in the research, factor analysis was performed on the scales used and dimension reduction was performed. The sample size must be sufficient to perform factor analysis. The sample size used in this study is 1130, which is quite goodhigh for factor analysis and other multivariate statistical analyses. While performing the factor analysis, when using the Varimax Rotation Technique, while obtaining the factors, the eigenvalue of the factor greater than one was taken into account. It has been stated that a factor load of 0.45 or above is a good criterion. Therefore, in this study, the lower limit threshold for factor loadings was taken as 0.45. Kaiser Mayer Olkin (KMO) values were obtained as 0.864 for the fear of FC-19S, 0.848 for the ON-R Scale, and 0.713 for the PSQI, and these values are evaluated as good and excellent in the literature^45^. As a result of the analysis, all questions were gathered under a single-factor structure as in the original scale of the FC-19S. The factor loadings of the expressions collected under this single dimension of this scale were found to be in the range of 0.66–0.85. The total variance explained was calculated as 63,579%. It is acceptable that the total variance explained in the social sciences is between 40 and 60%. As a result of the factor analysis for the ON-R Scale, a single factor structure was obtained for the scale. The ratio of explaining the total variance of the factors obtained was calculated as 55.2810%. Therefore, the explanation rate for the total variability of this factor is in the range of 40–60%^54^, which is accepted in the literature and is at a statistically significant level. The factor loadings of the expressions related to the subdimensions of the PSQI were found in the range of 0.60–0.88 and were considered high. The total variance explanation rate was calculated as 54.208%.

The relationship between fear of COVID-19, orthorexia nervosa, and sleep quality is shown in Table 3. As the fear of COVID-19 increased, orthorexia and the total sleep quality score increased. When orthorexia increased, the total sleep quality score increased. According to the correlation findings, there is a very weak relationship between fear of COVID-19 and orthorexia nervosa and a weak relationship between fear of COVID-19 and sleep quality. There is also a low correlation between orthorexia nervosa and sleep quality^55,56^.

**Table 3:** The relationship (correlation) between fear of COVID-19, orthorexia nervosa, and sleep quality.

Scales	FC-19S	ON-R	PSQI
FC-19S	1		
ON-R	0.073*	1	
PSQI	0.208**	0.197**	1

*p < 0.05, **p < 0.001.

When the regression analysis results for the model established for the relationship between fear of COVID-19, orthorexia, and sleep quality were examined, it was found that there were statistically significant differences in all of the variables. In this study, how significantly the independent variables in the established model predicted the dependent variables was evaluated by examining the regression coefficients. For this, the regression coefficients of the variables in the established model, presented in Table 4, were examined.

**Table 4:** Model characteristics for the mediation analysis.

Dependent variable		Independent variable	B	Standard error	Standardized coefficient β	t	P
(a) PSQI	←	FC-19S	0.089	0.012	0.208	7.144	< 0.001
(b) ON-R	←	PSQI	0.063	0.009	0.197	6.740	< 0.001
(c) ON-R	←	FC-19S	0.010	0.004	− 0.073	2.459	< 0.05

When the results are evaluated, a one-unit increase on the FC-19S scale causes an average 1% increase on the ON-R scale, while it causes an 8% increase on the PSQI. It was determined that the PSQI total score caused an average of 6% increase in the ON-R scale score. In addition to the overall effect of fear of COVID-19 on orthorexia nervosa in this model (path c; ß = 0.073, SH = 0.004, t = 2.459, p = 0.014 CI [0.002,0.001]), it has been concluded that it has both a direct effect and an indirect effect through sleep quality (Table 5). It was determined that sleep quality, the variable tested between the 95% confidence interval, lower and upper limits, fully mediated the relationship between fear of COVID-19 and orthorexia. In light of these findings, it was seen that hypothesis H4 was supported.

**Table 5:** Bootstrapped direct and indirect effects.

PSQI	B	Standart deviation	Confidence interval	
			Boot LLCI (Low)	Boot ULCI (High)
İndirect Effect	0.005	0.001	0.003	0.008
Direct Effect	0.005	0.004	− 0.003	0.013
Total Effect	0.010	0.004	0.002	0.018

## Discussion

In this study, the relationship between the COVID-19 fear levels and orthorexia nervosa tendencies of adult individuals under quarantine and whether the sleep quality of individuals has a mediating role in this relationship was examined. A low level of direct correlation was found between the level of fear of COVID-19 and the level of orthorexia. In addition, when the results were evaluated, a one-unit increase on the FC-19S scale caused an average 1% increase on the ON-R scale, while it caused an 8% increase on the PSQI. It was determined that the PSQI total score caused an average of 6% increase in the ON-R scale score. In addition, when other results were evaluated, it was determined that sleep quality caused a 6% increase in orthorexia, while a one-unit increase in fear of COVID-19 caused an 8% increase in sleep quality. There are also findings in the literature that support the present study results. In one study, it was determined that active people showed lower fear of COVID-19 and higher orthorexia than inactive people, and orthorexia was associated with psychological and physical health, explaining 7% of the effect on activity and FC-19S^16^. Another focus group study conducted during the pandemic found that individuals with eating disorders experienced worse sleep and worse mental health. However, it has been reported that healthy participants tend to recover and cope at a higher level^57^. Similarly, those who already had a chronic disease in themselves or any of their family members, as well as those who had weight changes during the pandemic, were found to have a higher orthorexia nervosa among the associated psychosocial factors in the current study. Although the prevalence of orthorexia differs between different sample groups in the literature, the majority of the studies supported the fact that the COVID-19 pandemic has exacerbated eating disorders or irregular eating patterns, namely, emotional and uncontrolled eating patterns^58^. Rodgers et al.^59^ focused on the three main causes of increased eating disorders during the pandemic and identified that the first cause was the reduced social activities resulting from social restrictions and prolonged lockdown, the second cause was the raised awareness of healthy nutrition and consumption of harmful food through social media, and the third cause was the restriction of food intake due to fear of COVID-19 through contaminated food. It can be thought that the fear of infections associated with food contamination and the pursuit of healthy food to have stronger immunity against COVID-19 increased the orthorexic tendency among the participants. Additionally, the present study revealed that during the pandemic period, the participants had a high level of fear of COVID-19, and their level of sleep quality decreased considerably. Other studies focusing on this topic have reported similar results. In a meta-analysis, it was determined that the pooled estimated prevalence of sleep problems in the general population during the pandemic was 37%^30^. In a Turkish study, 55.1% of the participants had poor-quality sleep^29^. Fu et al.^31^ studied people in Wuhan, China, during the pandemic and determined that 30% of them suffered from sleep disorders, largely due to anxiety and depression. Gupta et al.^28^ and Targa et al.^42^ both revealed in their studies that participants’ sleep quality worsened after the onset of the pandemic. They found that people were sleeping less at night, more during the day, going to bed, and waking up later than usual, and consequently, there was a significant correlation between negative mood and sleep quality. In another striking study on the subject, smartphone addiction increased during the pandemic period, which resulted in a decrease in the production of melatonin, which is considered an important hormone for the biological regulation of sleep, by smartphone light emitting short wavelengths. The study also found a significant correlation between insomnia severity indices^10^. Xiao et al.^60^ observed that as individuals became more socialized during the period of pandemic isolation, they slept better and became less stressed and anxious. In the present study, it was found that people with chronic diseases had poor sleep quality. An American-based study reported that out of 2,474 university students, 24.5% experienced high levels of stress during the pandemic, 31.3% suffered from clinical insomnia, and approximately 80% had poor sleep quality, as revealed by their PSQI scores^61^. In Jordan, a study found that out of 6,157 people, 76% had poor sleep quality. The same study also indicated that the lockdown had a negative effect on people’s mental health (62.5%), and women who had less income and were smokers during prolonged lockdown were at higher risk of developing sleep disorders and depressive symptoms^62^. Other studies reported that being female, already having a chronic disease and/or sleep disorders before the lockdown, and consuming caffeine were all predictive variables for high stress levels, insomnia, depressive symptoms, and poor sleep quality^34,61^. This is compatible with the findings of the present study, indicating that the fear of coronavirus is higher in those who are female and have chronic disease. Other studies on this topic also revealed that being in a risk group due to chronic diseases and having lost other family members due to COVID-19 caused people to fear COVID-19^63,64^. Being at risk of chronic diseases (e.g., heart disease, hypertension, and diabetes) was correlated with more problematic recovery—and even death—from COVID-19. The elderly and people with chronic diseases made up the bulk of COVID-19-related deaths, which in turn exacerbated people’s fear of it. Several researchers found that fear of COVID-19 was greater among women than men^33,65^. They moreover attributed that to the fact that women are more sensitive and vulnerable than men. The present study revealed similar results. It was believed that it was socially more acceptable for women—as opposed to men—to express their fears about COVID-19. This was caused by gender roles, such as being strong and brave for men. It was also found in the study that the participants who fell into a low-income bracket were also more afraid of COVID-19. At the root of that are the changes in work conditions during the pandemic. Adams-Prassl et al.^66^ reported in their study that the pandemic forced 57% of the subjects to take on minimum wage jobs, 8% to lose jobs, 33% to be at risk of losing their jobs, 35% to expect to earn less money as time proceeds, and 49% to be worried about expensive bills. In other words, there was a correlation between fear of COVID-19 and low-income background. An Indian study reported that the majority of subjects with chronic diseases (e.g., diabetes and hypertension) faced numerous economic and psychosocial challenges during the pandemic, including job and income loss and lack of access to health services and medication^67^. Based on these results, poor sleep quality may be associated with increased anxiety and stress, which can exacerbate the effects of fear of COVID-19 and contribute to the development or worsening of orthorexia nervosa. It is possible to explain these relationships based on the self-actualization theory of Abraham Maslow, the leader of humanistic psychology. In his theory, hierarchical needs are classified into five basic categories of needs: physiological, safety, love, esteem, and self-actualization. Requirements such as adequate food and sleep, which are among the physiological needs, include the body's efforts to meet homeostasis needs and are vital^68^. These, on the other hand, constitute the needs of the individual who is interrupted first in disaster situations such as pandemics. From this perspective, ensuring good sleep quality during difficult life events can help alleviate the effects of fear of COVID-19 and reduce the risk of developing orthorexia nervosa or its symptoms. Therefore, it is recommended to conduct further studies to compare the correlations between the tendency toward orthorexia nervosa, sleep quality, fear of coronavirus, and biopsychosocial factors in different sample groups. It is critical that medical staff take the necessary precautions on these issues.

## Conclusion

Based on the study's findings, there were significant connections discovered between fear of COVID-19, orthorexia nervosa, and sleep quality. Individuals with orthorexia often have strict and unchanging eating habits, and stress can make it challenging to maintain these patterns. Additionally, sleep issues correlate with higher levels of psychological distress, such as depression and anxiety. Therefore, it is crucial to examine gender, stress, anxiety, social media, eating habits, and cultural norms as comparative and longitudinal factors in very low sleep quality groups in future studies. Healthcare providers have a critical role in designing and implementing programs that can help society better handle acute situations such as pandemics, support biopsychosocial aspects, and improve resilience.

### Strengths and limits

Among the strengths of the study is the ability to reach large groups of environments (1130) and capture previously unexamined variables during a time of restrictive practices such as the quarantine period due to COVID-19. One limitation of this study was that it was conducted via an online survey. Thus, it did not reach out to those who did not complete the survey. A second limitation is that the results are limited to self-reports and therefore cannot be generalized for all of society. Because the measurements related to sleep and orthorexia are based on self-reporting, the inability to use more objective measurement tools (actigraphy and polysomnography, etc.) is an important limitation. In addition, the number of subjects of the study (1130) was not convenient to make such applications. The biggest limitation of our study is that the gender effect and sleep quality level could not be controlled. The majority of the participants were women, and studies on adults indicate that women experience more sleep problems^34,69^. The low sleep quality can be explained by the fact that the majority of the participants were female.

## Consent to participate

Written informed consent was obtained from all individual participants included in the study.

## Data Availability

The dataset analysed during the current study is available from the corresponding author upon reasonable request.
